# A Smart Technology Intervention in the Homes of People with Mental Illness and Physical Comorbidities

**DOI:** 10.3390/s23010406

**Published:** 2022-12-30

**Authors:** Cheryl Forchuk, Abraham Rudnick, Deborah Corring, Daniel Lizotte, Jeffrey S. Hoch, Richard Booth, Barbara Frampton, Rupinder Mann, Jonathan Serrato

**Affiliations:** 1 Lawson Health Research Institute, London, ON N6C 2R5, Canada; 2 Arthur Labatt Family School of Nursing, Western University, London, ON N6A 3K7, Canada; 3 Department of Psychiatry, Dalhousie University, Halifax, NS B3H 2E2, Canada; 4 Nova Scotia Operational Stress Injury Clinic, Nova Scotia Health Authority, Dartmouth, NS B3B 1Y6, Canada; 5 Department of Computer Science, Western University, London, ON N6A 3K7, Canada; 6 Department of Epidemiology & Biostatistics, Western University, London, ON N6A 3K7, Canada; 7 Department of Public Health Sciences, University of California Davis, Davis, CA 95616, USA; 8 Ontario Peer Development Initiative, Toronto, ON M5S 2R4, Canada

**Keywords:** smart technology, smart homes, mental health, eHealth, physical health

## Abstract

Appropriate support in the home may not be readily available for people living in the community with mental illness and physical comorbidities. This mixed-method study evaluated a smart home technology intervention for individuals within this population as well as providing health care providers with health monitoring capabilities. The study recruited 13 participants who were offered a smartphone, a touchscreen monitor, and health devices, including smartwatches, weigh scales, and automated medication dispensers. Healthcare providers were able to track health device data, which were synchronized with the Lawson Integrated DataBase. Participants completed interviews at baseline as well as at 6-month and 12-month follow-ups. Focus groups with participants and care providers were conducted separately at 6-month and 12-month time points. As the sample size was too small for meaningful statistical inference, only descriptive statistics were presented. However, the qualitative analyses revealed improvements in physical and mental health, as well as enhanced communication with care providers and friends/family. Technical difficulties and considerations are addressed. Ethics analyses revealed advancement in equity and fairness, while policy analyses revealed plentiful opportunities for informing policymakers. The economic costs are also discussed. Further studies and technological interventions are recommended to explore and expand upon in-home technologies that can be easily implemented into the living environment.

## 1. Introduction

Smart homes to support mental illness are an appealing new concept but have not yet been implemented at the community level as a form of mental health care or support. The iterature reviews have found that hospitalizations, the burden on care providers, consultation and wait times, and healthcare costs can all be reduced through health monitoring technology [1]. Although new technologies are rapidly developed for the consumer market, many are not stringently tested in a mental health care setting, leading to a lack of robust literature. This rapid pace of development also means that new technological advances become obsolete quickly over time [2].

Mobile technologies such as smartphones have been utilized to support mental illnesses such as bipolar disorder [3] and schizophrenia [4]. Patient beneficence and well-being can also be supported using smart technology by providing greater access to information and resources, as well as through symptom tracking and monitoring features [5]. A systematic review of smart technology for mental illness detailed that smartphone applications were the most studied but monitoring and adherence supports were lacking significantly [6]. A more recent systematic review also revealed that research into technological interventions based on the Internet of Things model is still lacking, with mental health data scattered and segregated depending on the vendor/platform for these devices [7]. Smart technology intervention should require minimal input and provide continuous monitoring passively that does not interfere with daily activities.

Physical activity can also be an indicator of changes to mental health and/or indicative of a crisis. Including physical activity in daily routines can be difficult, particularly as symptomology, motivation, experience, fatigue, and poorer access to resources are common considerations for people with mental illness [8]. This is particularly pertinent during the current time, with physical activity and mean peak heart rate readings are decreasing significantly during COVID-19 lockdowns [9]. As such, the use of smart technology to maintain a healthy lifestyle is even more needed, particularly among vulnerable individuals who may experience barriers due to their health status, lack of accessibility, or socioeconomic status.

Two early inceptions of the current study, each lasting 12 months, attempted to provide support systems within an individual’s environment in hospital settings and transitional hospital apartments [10]. The prototype intervention provided in the hospital setting was largely successful, with devices and software communicating and funneling data to a centralized database as planned. Upon successful testing, the intervention was then prepared for the community setting. This study, outlined at the ICOST 2022 conference, set out to establish the use of smart technology in assisting individuals with mental illness and physical comorbidities living in housing provided by the Canadian Mental Health Association (CMHA) and London-Middlesex Community Housing (LMCH) [11] (pp. 86–99). The objective of this study was to establish and evaluate smart home technology in the community to assist people with mood and psychotic disorders. We hypothesized that the introduction of smart technology would: (a) increase levels of community integration; (b) increase housing stability; (c) decrease health, justice, and social service utilization; and (d) support mental and physical health.

The study also sought to answer the following research questions: (a) What are client and staff experiences of smart mental health homes? (b) How do clients and staff perceive the utility of smart technologies in the home? (c) What improvements to the technologies do they suggest? (d) What ethical issues are identified with the use of smart mental health homes? (e) What are policy issues identified with the use of smart homes? (f) What commercialization are issues identified by key stakeholders in relation to smart homes? These questions seek to not just evaluate the current study but also to inform future iterations of the intervention provided.

## 2. Materials and Methods

### 2.1. Design

This study used a within-group, mixed-methods, repeated-measures design. Interviews were conducted over three assessment time points conducted at baseline (Time 1), 6-month (Time 2), and 12-month (Time 3) follow-ups. The assessments included an individual interview with each participant. However, due to the COVID-19 pandemic, interviews and focus groups were switched to virtual and telephone formats, and focus groups were conducted via a one-to-one discussion instead to maintain safety as well as convenience for participants. Ethics approval was obtained through Western University’s Research Ethics Board.

### 2.2. Sample

The research team first recruited healthcare providers who then referred potential participants for the study. Participants from a range of housing types were eligible to take part. The inclusion criteria for participants were as follows.

1. Must be on a care load of a participating health care provider.
2. Able to understand English to the degree necessary to participate.
3. Living in housing provided by the CMHA or LMCH.
4. Diagnosed with a psychotic or major mood disorder.
5. Must be between the ages of 18–85 years old.
6. Able to provide informed consent.

CMHA workers of the participants were also recruited as part of the study in order for them to provide their perspectives and guidance on smart technologies.

### 2.3. Intervention

This study consisted of two interactive platforms, the Lawson Integrated Database (LIDB) and the Collaborative Health Record (CHR). The LIDB is an information management platform that collates and manages health data and is protected behind the St. Joseph’s Health Care hospital firewall [12] (pp. 131–142). It is a web-based application that can be accessed by healthcare professionals and research staff via secure log-in. The LIDB connects to the clouds of health monitoring devices and is able to automatically export data from each cloud with encrypted authentication keys and SSL connectivity. All data were segregated within its own database schema and presented through tables, graphs, and text-based outputs. Incremental data backups were performed nightly, and full backups were performed weekly with all data encrypted to maximize data security. Both nighty and weekly backups were stored securely off-site as per LIDB protocols. The LIDB also utilizes virtual servers to move from one server to another to enable continuous operations with no impact on users. Prompts and reminders such as appointments or medication reminders can be programmed easily within the LIDB by healthcare providers to appear on screen devices at a set time and date. These were sent either as an email or SMS text message to the smartphones or appeared on the touchscreen monitor.

The CHR allows healthcare providers to send and receive questionnaires from clients and to track progress over time. It sends questionnaires (“Qnaires^®^”), both standardized or customized, as an SMS text message, email, or both to participants. The link then opens to a custom webpage with the questionnaire for participants to complete. Should the participant indicate a potential crisis, such as suicidal ideation, an alert is sent to their care team. Data from Qnaires^®^ were then backed up on the platform, and progress can be monitored over time. This allowed for comprehensive workflows among a diverse group of healthcare providers who can focus and respond to specific symptomology or participant needs. The platform has the additional feature of videoconferencing and instant messaging, which is important if Qnaires^®^ responses suggest further attention.

Screen devices offered included smartphones (Samsung J3^®^) and wall-mounted touch-screen computer monitors powered by a Raspberry Pi-3 B+^®^ mini-computer. The latter was developed by the research team programmer, who also designed and customized the interface. These devices received and responded to prompts from the LIDB to assist participants experiencing cognitive deficits and to facilitate self-care. Participants were able to “acknowledge” prompts and reminders on the touchscreen monitors by pushing a “Got It” button on the screen, which sent an automated email to the care providers who set that reminder. Additionally, a button that says “Please get in touch with me” was included, which sent a message to the participant’s care team requesting them to schedule a meeting. When pushed, a prompt notified the participant that this was not a crisis button and to contact a crisis line if they needed immediate support.

Health monitoring devices that were offered to participants included a Withings-Nokia Body+^®^ smart weigh scale, a Fitbit Charge 3^®^ activity tracker, and a Karie^®^ automated medication dispenser developed by Ace Age. The activity tracker provided the monitoring for heart rate, steps, and sleep quality. The automated dispenser allowed participants to self-medicate (if appropriate) and reduced the need for participants and/or their healthcare providers to collect medications from their pharmacy. Apps for each of these devices were added to the participants’ smartphones so that they could also track and monitor their own data. Although specific activity logs for device usage were not specifically recorded, healthcare providers could observe the measurements provided by the health devices through the LIDB, thereby providing an idea as to the frequency of device usage.

**Figure 1:**
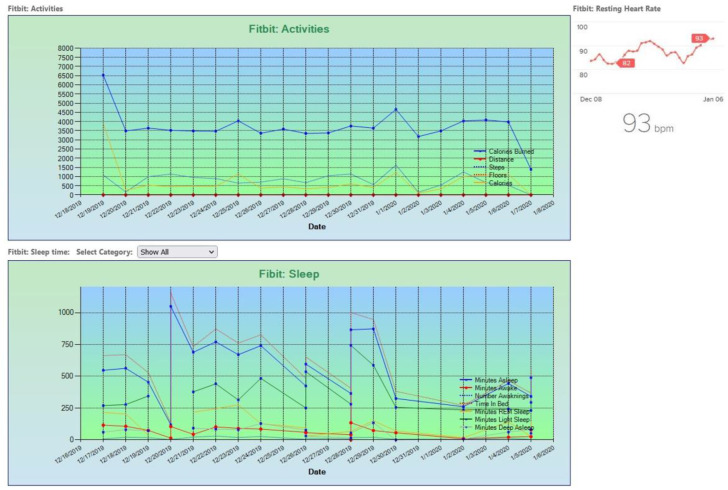
Raw Data Output within the LIDB.

Care providers were able to securely log into the LIDB and view data from their participant’s health monitoring devices (see Figure 1). Both LIDB and CHR, they were able to send reminders and messages to the screen devices. This bidirectional approach allowed care providers to respond to the health data and communicate prompts, directions, or queries with the participant.

### 2.4. Procedure

The research team met with prospective participants, and after obtaining informed consent, participants selected the equipment they felt they needed and completed the baseline interview. Interviews were conducted one-to-one with a member of the research team either in-person or virtually. Equipment selections were then verified and approved by the participant’s healthcare provider before being installed by the research team’s programmer. Training on the equipment was provided by the research team programmer as well as the research coordinator. Technological literacy was measured at baseline through the demographics measure of the interview. Healthcare providers also received training on how to use the CHR and LIDB from the research coordinator. Healthcare providers also received training with the devices in order to provide immediate troubleshooting on-site should a participant experience any difficulties. The research coordinator contacted the participants monthly to check in on any potential technical difficulties and offered assistance if issues arose. Calls and emails were also made to healthcare providers to offer support and troubleshooting with the LIDB and CHR, as well as any general concerns regarding the devices.

Focus groups with participants and healthcare providers were conducted separately to prevent one group from influencing the other. These were conducted in a group format prior to the COVID-19 pandemic but then switched to virtual groups via telephone or teleconferencing software. Both participants and healthcare providers were also offered the opportunity for a one-to-one discussion. All focus groups and one-to-one discussions were recorded and then transcribed verbatim by a trained member of the research team.

### 2.5. Instruments

The following assessments were utilized for the semi-structured interviews conducted with participants: Community Integration Questionnaire-Revised (CIQ-R) [13], Short-Form 36 (SF36) [14], EuroQol-5 Dimension-3 Level (EQ-5D-3L) [15], the Health, Social and Justice Service Utilization (HSJSU) questionnaire [16], and a Perception of Smart Technology Questionnaire. The last was a researcher-developed measure that assessed participants’ attitudes and opinions of the devices provided. Demographic and housing history data were also collected during each interview. Open-ended qualitative questions regarding the use of the devices and software platforms were utilized during the focus groups and one-to-one discussions.

### 2.6. Data Analysis

The participant’s health, housing, service utilization, and community integration were assessed using the instruments. Descriptive statistics are provided for the outcome measures. Due to the small sample size, comparative analyses across time points were not possible, and generalization beyond the sample was limited. Qualitative data were analyzed using a thematic ethnographic approach [17] (pp. 33–72) to ascertain the collective experiences and implications of responses. Data collected from the health devices and the Qnaires^®^ were not obtained for analysis by the research team in order to maintain participant privacy and not to interfere with the patient-provider therapeutic relationship. As part of a standardized evaluation framework for smart technology and mental health [18], ethical and policy analyses were conducted to explore ethical implications as well as potential opportunities for policy change.

## 3. Results

A total of 13 participants were interviewed at baseline. Table 1 summarizes the demographic characteristics of the sample. Nine participants completed the full 12-month study; two passed away due to natural causes before Time 2. At Time 3, one declined to complete the interview, while the other participant could not be reached. No participants experienced housing instability throughout the course of the study. Two participants were eventually moved from group homes to independent apartments. The vast majority of participants were single or never married (*n* = 10), indicating that these participants may need additional support if they are living alone.

**Figure 2:**
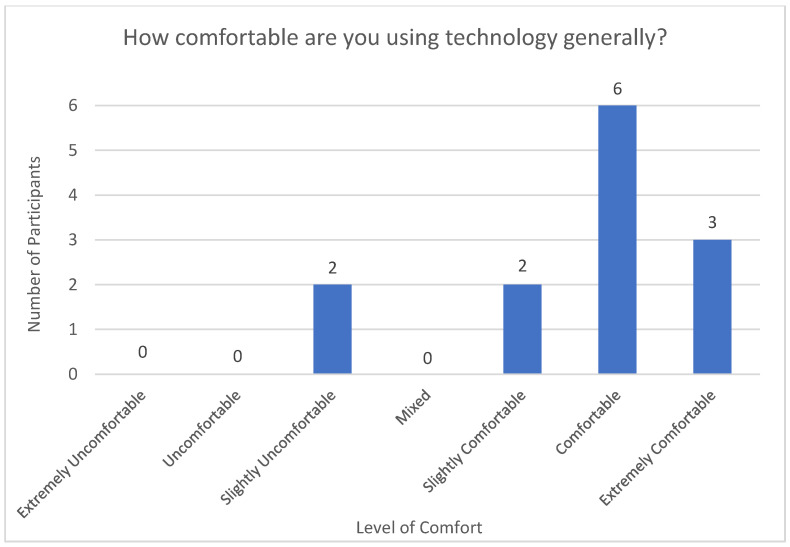
Level of Comfort with Technology at Baseline.

In terms of technological literacy, the participants were asked at baseline to score their experience with devices (see Figure 2). The sample overall indicated they were comfortable with technology. Participants largely rated that they were comfortable with technology in general. Three participants reported they were “extremely comfortable,” six reported they were “comfortable,” and two participants each reported they were “slightly comfortable” and “slightly uncomfortable,” respectively.

**Figure 3:**
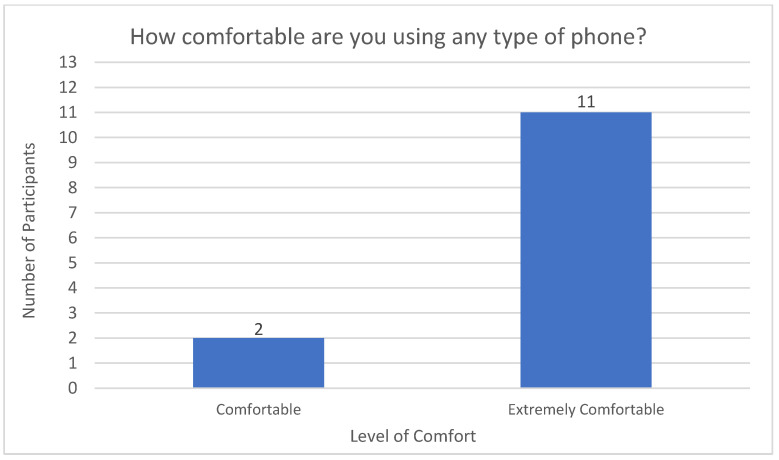
Level of Comfort with Phones at Baseline.

Smartphones were an integral aspect of the intervention as this allowed them to send and receive SMS text messages and/or emails with notifications, conduct videoconferencing appointments, complete questionnaires, and track their health data using the apps for each health device they selected. Participants were also asked about their comfort level in using smartphones (see Figure 3) to assess literacy as well as identify individuals who may need extra support and training in using their phones. The overwhelming majority of participants (*n* = 11) reported they were “extremely comfortable” with using their phones. Two rated their comfort level as “comfortable.” No participants rated their use of phones as less than comfortable.

### 3.1. Quantitative Findings

#### 3.1.1. Community Integration Questionnaire-Revised

The CIQ-R is a measure used to examine the degree to which each individual participates in communal, vocational, and home activities [13]. A total score is obtained by summing the four subscale scores, with higher scores indicating a greater degree of integration. The mean scores on all CIQ-R subscales and CIQ-R Total scores across all three time points are reported in Table 2. The CIQ-R asked participants how often they completed certain activities and who else completed these activities with them.

#### 3.1.2. Short Form 36

The SF-36 is a self-reported measure of health status consisting of eight multi-item subscales assessing physical and mental health phenomena [14]. Subscales are scored on a scale of 0–100, with higher scores representing better health outcomes. The mean scores on all SF-36 subscales across all three timepoints are reported in Table 3.

#### 3.1.3. EQ-5D-3L

The EQ-5D-3L is a measure of the quality of life which asks individuals to report their health state at the present time [15]. The Visual Analogue Scale revealed participants’ mean self-report ratings of their overall health, physical health, and mental health from Time 1 to 3 on a scale of 0 (lowest health) to 100 (best health) (see Table 4). Participants gave their ratings based on how they felt at the time of the interview.

#### 3.1.4. Health, Social, and Justice Service Utilization

The HSJSU is a scale that measures an individual’s utilization of community and hospital services type, frequency, and reason for using these services [16]. A variety of health and social services were accessed throughout the study. The HSJSU defines a health provider as an individual who provides support for a health concern, such as a doctor, nurse, or psychiatrist. A social services provider is defined as an individual who provides support with housing and finances. This included social workers, housing workers, and justice workers such as probation officers. The HSJSU asks participants about accessing these services in the past month prior to the interview. Other services accessed in the past 6 months prior to the interview are investigated. Table 5 illustrates the utilization of these services.

It should be noted that one participant at Time 2 reported calling a crisis line every day for the past 6 months and estimated 180 calls. For each instance, the participant reported that they were subsequently visited by a crisis team, who followed up on each call.

At Time 1, there was one participant who reported being visited by a crisis team. Despite the interviewer’s best efforts to ascertain the number of visits, the participant was unable to provide a number of instances as they stated they did not know how many visits had occurred. As such, this is represented as a dash in Table 5.

#### 3.1.5. Perception of Smart Technology

**Figure 4:**
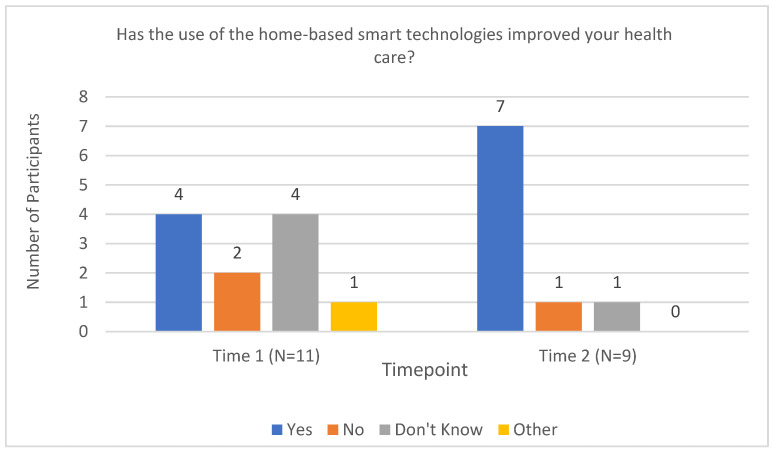
Perception of improvements to health care following the use of smart technologies.

**Figure 5:**
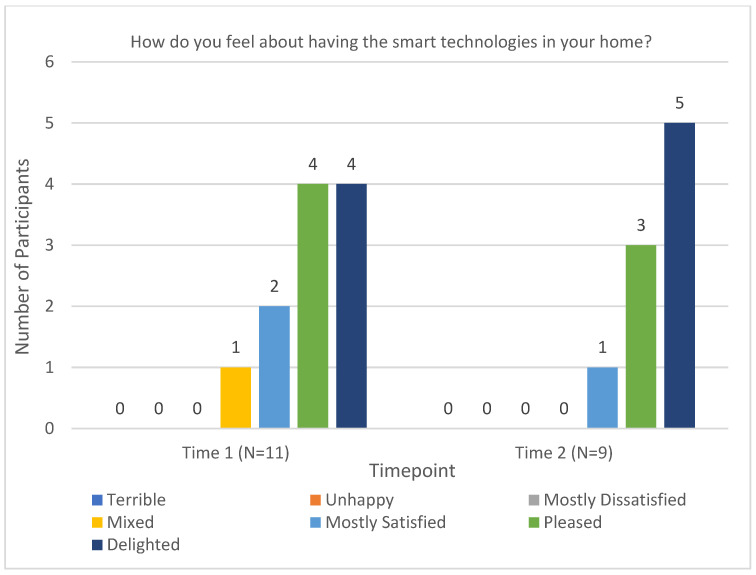
Acceptability of smart technologies in the home.

The Perception of Technology questionnaire was a researcher-developed measure that intended to inquire into participants’ attitudes and opinions of the equipment provided to them. Four key questions from the questionnaire are reported below. The first inquired as to whether participants felt the technologies improved their health care (see Figure 4). This was asked at Times 2 and 3 after the technologies had been implemented in their homes. The second question focused on the acceptability of the technologies in the home (see Figure 5). The majority of participants responded favorably at Time 2—Mixed (*n =* 1), Mostly Satisfied (*n =* 2), Pleased (*n =* 4), and Delighted (*n =* 4). At Time 3, there was a slight increase in positive ratings—Mostly Satisfied (*n =* 1), Pleased (*n =* 3), and Delighted (*n =* 5).

The third key question focused on recommendations for what devices should be added to future interventions. The suggestions were evenly spread, with a blood pressure cuff receiving the most recommendations (*n =* 2), followed by a tablet, a smart television, a smart glucometer, a Google Home device, and a newer model of Fitbit (all *n =* 1). However, the most frequent answer was “Nothing else/None” (*n =* 3), as participants felt the devices offered as part of this study provided suitable coverage for their needs.

The fourth question investigated the devices that were most used and the devices that were least used (see Table 6). The Fitbit was the most used device, owing to the fact, as participants stated, that it provided valuable and interesting data that participants liked seeing in terms of their health and did not require as much interaction as the other devices. The touch-screen monitor was reported as the least used device by participants. Participants reported during focus groups that the monitor would experience technical glitches and preferred the smartphone as a screen device. Furthermore, some healthcare providers did not use it as frequently for displaying prompts and reminders. The smartphone provided participants with greater contact with a wider circle of care (i.e., doctor, psychiatrist).

#### 3.1.6. Economic Costs of the Intervention

The full upfront cost of the intervention, with all devices, phone plan, and Wi-FI connectivity, came to a total of $12,100 for the initial year. As the devices would already have been purchased, the subsequent years of using the intervention would be significantly less, and only the phone and Wi-Fi bills would need to be paid. This study used a low-cost Wi-Fi plan for tenants of non-profit housing agencies and a discounted phone plan totaling CAD$210 per year.

Based on in-house data at the institution of the principal investigator, a hospital bed with the necessary staffing needs costs an average of CAD$638 per day for a total of CAD$232,780 per year. This cost does not include the provision of any personal devices. The cost of the intervention is equivalent to two days in a mental health institution. As the intervention is designed to provide support in the home, we would highlight that the potential cost-saving of keeping people at home instead of being hospitalized is significant.

### 3.2. Qualitative Findings

#### 3.2.1. Physical Health Benefits

Several themes pertaining to healthy living were discussed by the participants. The majority of participants (*n* = 5) noted at the study end that they were motivated to be healthier through exercising and maintaining a healthy weight by utilizing the Fitbit and the weigh scale provided to them. The health data on the apps on their smartphones allowed them to track their progress which provided a level of accountability.

*The scale especially um it, it, it allowed me to keep track of what was happening as far as weight and things like that went and um yea it didn’t, it has motivated me um to start watching my diet and things like that.*

*Increased. It increased my exercise. It made me drink more water. It made me just move for no reason. Um, because of the reminders to move, be like, “Oh, you know it’s 12:50, you’ve done five steps this hour”, you know?*

Healthcare providers (*n* = 6) also reported that their clients were becoming more motivated to use the devices provided to lead healthier lifestyles and, in some cases, assist with symptoms of pre-existing physical conditions.

*Uh, I definitely noticed, um, that she was able to maintain a healthy lifestyle, um, in term, or I guess a healthy weight. There was, you know, only small fluctuations in their weight, but it was nice to see that. Cause I know they, they talk about that a lot. Um, just wanting to, because they have another, um, illness related to their weight, like polycystic ovarian syndrome. So they were, it was more of like an important thing to focus on managing or getting, you know, getting to be a healthy weight so that the symptoms of that are minimized.*

As a result, participants (*n* = 6) noted that they noticed benefits to their physical health. Monitoring and tracking health data were seen as important advantages of the devices in support of weight loss and more activity than usual.

*No, it’s my mobility decreased a bit, due to exacerbated symptoms, physical symptoms and stuff. So I am using the walker more but even still. With being able to do the exercises and stuff with help from the Fitbit. I have more range of motion, less general, high pain, days, less days where I spend all day in bed and not do anything at all and not move and just sleep.*

#### 3.2.2. Mental Health Benefits

Other participants (*n* = 7) noted benefits to their mental health through this new ability to be healthier and through the cognitive support the technology offered.

*I have issues with memory and being able to have reminders to drink water. Um, how, cause instead of feeling more lethargic and fatigued from not drinking water, um, I felt less lethargic, less fatigued. Therefore, it increased my mood because I wasn’t sleeping all the time. And for me, a trigger for depression is sleeping all the time.*

It was noted that the technologies were also able to support mental health through a biofeedback approach with the devices monitoring physical activity.

*…and like as I say the, the Fitbit, especially the pulse um the heart rate um is, is really helpful because um if I’m having problems with my mental state sometimes, I need to look at, especially with anxiety, I need to look at my pulse and, and sort of be aware of it and help, it can help me bring it down…*

Healthcare providers also discussed that mental health was also supported through the provision of enhanced communication (*n* = 3). During the COVID-19 pandemic, when isolation was required, support was harder to access, and so devices such as the smartphone were able to bridge this gap.

*Um, you know, I think that really is also another layer to, you know, one’s mental health, right. Being able to stay in contact with people you care about. So I think that that definitely had some benefits, um, especially during COVID, um, I’ve been also, you know, like.*

Enhanced communication was also seen as a major benefit of the study. Participants (*n* = 6) noted they were able to maintain communication with their friends, family, and healthcare providers during the COVID-19 pandemic. Not only did the greater communication provide support in terms of social activity and reducing isolation, but it was also reported to support mental health through accessing additional resources.

*Um, well being connected with your family definitely helps you with your mental health and like being able to follow-up with appointments and stuff like that. Um, yeah and I also have been able to like, find resources about my mental health like on CAMH and stuff like that and um meditation resources as well.*

*Because. well it’s important for mental health and stuff like having, having the friends and people to talk to instead of being like alone having, having nobody.*

Healthcare providers (*n* = 5) also spoke of the social benefits of providing participants with a smartphone but also noted that the technology-enhanced communication with the care providers as well as flexibility in communicating with them.

*…Like some clients especially like with mental health, they find it easier for them to, instead of talking to someone over the phone, to text and communicate that way.*

#### 3.2.3. Cognitive Benefits

There were numerous instances of participants (*n* = 3) noting that the reminders and prompts from the technology provided them with the support needed to maintain healthy lifestyles and live safely in their own homes. These assisted participants with memory issues and helped them with organizational skills such as attending appointments, activities of daily living, and taking medications at specific times.

*…there’s also tips on the app that you can get sent to your Fitbit. And some of those are great too, you know, like I get water reminders a few times a day, or, you know, “Hey, it’s time for lunch. Remember, drink a glass of water with lunch.” And with that, I started drinking more water with my meals.*

In particular, the two participants with the medication dispenser noted that they no longer missed doses.

*Having the ability to monitor um the, the medication was a problem and the technology just sort of took that problem away because it replaced the need for me to keep track of things… I like the med dispenser because it helps me with keeping on track of taking my meds, with compliance.*

*The med machine was huge, the biggest one, probably the only one that I really, really benefitted from because I’m, I was on my meds consistently.*

#### 3.2.4. Technical Difficulties

With technological interventions, there are also some limitations and barriers to overcome, such as technical difficulties experienced by participants (*n* = 5). One such issue is device reliability. There were some issues regarding difficulties with connectivity.

*Yeah, um, but when I switched phones it was very difficult to get um, to get my Fitbit to sync over Bluetooth.*

There were also concerns stated by participants regarding the accuracy of the device’s readings.

*There was a period of like 2 months where um it [the weight scale], it showed me as 30 pounds lighter than I actually was. So I had to like erase all that data and um, and that like, up until the day that happened and then it went back to normal.*

A precipitating factor for technical difficulties could be the lack of technological literacy and understanding of how specific devices work. Although comfort with technology was scored quite highly at baseline and monthly refreshers were provided by the research team, healthcare providers (*n* = 5) highlighted that frequent retraining would be recommended.

*I think there was, um, a few items that like the clients weren’t really familiar with or like, knew how to use our troubleshoot, even though like, I don’t know, maybe I’m just the younger generation. I know how to use it. Like the Fitbit. They don’t really know how to like sync it or look at their data on their, on the app, on the phones and stuff.*

Despite the technical difficulties and potential technical literacy concerns, healthcare providers (*n* = 2) also reiterated that mental illness may influence literacy and acceptability of smart technologies.

*What might come natural to some of our clients, because some of, many of our clients will feel insulted too. For some, it will be really struggle, probably not understand right. Uh at the same time um I’m pretty sure you guys noticed with some of the clients, paranoia’s a big key and doesn’t matter what your best intentions are, they’re always questioning what you’re trying to do.*

#### 3.2.5. Further Considerations

It is also recommended that alternative devices are available for participants who require additional assistance with using technology.

*Like I said, I have hand tremors, so takes me awhile to get it to work.*

*Also for a lot of people, including myself, I have actually had to, um, change what the clock looks like on, um, the Fitbit because the writing was too small and because I couldn’t read it and I, it, it, um, because my vision is bad, I actually have to keep the brightness on the highest about 90 some odd percent of the time. Because I can’t read it when it’s darker.*

In terms of future improvements for commercialization, the healthcare providers were largely pleased with the intervention. Simplifying the approach was recommended, with all data streamlined to one location. Although the LIDB was mostly used as the central hub, having two platforms along with the CHR required more attention. A single integrated database that performed the tasks of both platforms and is offered as an app was preferred.

*You’ll have to be streamlined through a maybe single-handed maybe app? … that will probably be uh better, for future reference, and it could be uh quite heavily used then. Or you may need the only thing, once it’s approved, I know it’s now in uh, in uh research base, in a research lab, you can’t really uh you know push harder than that. But I would say that had we had an app and that was the only app you can use to maybe, once our clients are approved and everybody uses the same app, that is streamlined and uniform, that would probably be much better yes. But otherwise, we really didn’t have much of a use of these health records, no.*

#### 3.2.6. Ethics Analysis

This project’s findings suggest that the use of the technologies advances equity and fairness by increasing access to care for people experiencing mental illness and physical comorbidities. The majority of participants at Time 3 stated that the devices improved their health care from within their own homes, suggesting access to support was improved beyond seeing a healthcare professional or admission to a hospital. During the COVID-19 pandemic, participants were able to access care from the safety of their own homes, many of which used the technologies provided by the study. Acceptability of the devices was found to be high, indicating that there were no significant concerns regarding the use implementation of these devices in the home. Another ethical advantage of this project was that it enhanced the autonomy of participants, reducing their dependence on some health services, such as by using the medication dispenser device. The qualitative analyses also revealed improvements in communication and accessing resources which could indicate a further enhancement to health equity. A caveat is that such reduced dependence did not occur for some participants, particularly those who were inexperienced with technology and hence required human support to use the technology. As noted in the qualitative analyses, technical difficulties and literacy was a challenge for some participants. Overall, this project’s findings are promising from an ethical perspective and suggest the need for larger-scale research on such technological interventions.

#### 3.2.7. Policy Analysis

It was felt that it would be purposeful to propose an amendment to the Assistive Devices Act in Ontario in order to incorporate smart technologies as assistive devices for mental health. These devices could also provide benefits to physical health, with participants in this study reporting that they were motivated to exercise more and live healthier lifestyles. As noted in our Qualitative and Ethics Analyses, the devices helped to support autonomy by facilitating participants to have greater control of their own living situation. Participants were able to maintain medication adherence, maintain a healthier lifestyle, communicate to a greater degree, and access resources for mental health. Devices aimed at supporting mental health are not currently covered by the Assistive Devices Act in Ontario, Canada. However, as this study demonstrates the feasibility of a tailored intervention using commercially available devices that can be replicated, it could be argued that policies in other countries and jurisdictions could also be informed. At the present time, individuals with mental illness or agencies supporting this population would have to purchase the devices themselves. For individuals in other jurisdictions, amending the current policy to provide funding for technological innovations in the home to support mental illness would be advantageous. Ultimately the goal for policy change would be to provide funding or coverage for the provision of needed devices for this vulnerable population that is proven to be beneficial, such as those used in this study.

## 4. Discussion

Smart technology interventions, such as this study, should be designed to provide support for a range of physical and mental health conditions [19]. This study positions itself as a foundation for future research among individuals with mental illness to build upon and expand further as feasibility has been established. The evidence from this study supports the notion that the research community should be designing and running larger studies to learn how these can be best tailored to the contexts and populations where they are needed. As there was a small sample size, it would be difficult to argue that this intervention represents a definitive approach to mental health care, but it provides an impetus for new research and begins to cover some of the gaps currently in the literature. Drastic changes in healthcare measures were not observed, which, although likely as a result of the small sample size, creates a further need for future large-scale research. Studies exploring mental and physical health interventions using an Internet of Things innovation are lacking. The study demonstrated that a technological solution is feasible by adopting an Internet of Things approach and bidirectional monitoring. The findings of this study also suggest that a technological intervention should not necessarily be focused solely on younger generations. Data from the Perception of Smart Technology questionnaire supported this in that the technologies were well-accepted and used by participants of different age groups. As the majority of participants were single or never married, this intervention may be needed support for individuals living alone without immediate assistance. The connectivity of the intervention with care providers could help to mitigate potential risks that may arise due to isolation or a lack of communication.

A key strength of this intervention was the use of non-clinical devices that are accessible to the public. Data from the clouds of these devices were able to be transmitted and collated in one database, the LIDB. Healthcare providers were able to access all the data in one location as opposed to individual databases or independent datasets. This allowed for a tailored approach where each participant could have a customized intervention. Participants were also able to connect their smartphones (either their own or one provided as part of the study) to their devices and access the data themselves using the devices’ apps. As this study focused on mental health, an implication for future research is to measure the effects of smart technology among individuals with physical health conditions. It would be highly beneficial for potential researchers to track health data (i.e., weight, steps, activity, heart rate, etc.) empirically to assess the impact on health behaviors and physical condition. There is also the opportunity to utilize this intervention to support physical conditions that may benefit from activity and weight tracking and notifications. This study found that smart technology helped to promote healthy lifestyle choices. Based on the qualitative findings, the devices acted as an accountability tool which further provided encouragement and motivation for healthier living. Frequent observation and self-monitoring of health data using personal digital assistants and daily feedback messages have been linked with weight loss in a previous study [20].

A systematic review by Liu et al. [21] reported there is no evidence that technology tracking and monitoring biometric data resulted in improvements to the quality of life or disability. However, the qualitative findings of this study suggest that the feedback from the health adjunct devices and their respective apps on smartphones can be helpful. The use of an activity tracker to encourage physical activity and exercise may have been the most contributory piece of equipment in this regard. Participants in this study reported how the technologies supported mental health, such as mood and anxiety, through greater communication and the ability to monitor physical health more easily. Cognitive support was also described as assisting participants in remembering tasks such as taking and tracking medications. There was some discussion in the qualitative analyses with participants that the weigh scale and the activity tracker may have occasionally given inaccurate readings. Future studies would be advised to frequently check devices for accuracy during the course of the study and allay participant concerns. The technician for the study was able to collect the devices for recalibration as and when needed, but assistance on-site was not always permissible due to the COVID-19 pandemic.

Based on the policy analyses, a key implication of this study could be the establishment of a multi-sector forum to advance this initiative of smart technology supporting mental health. This forum could include mental health advocates, researchers, policy-makers, and technology innovation experts to collaborate and develop new initiatives that support the current needs of people with mental illness. This would be beneficial as new tailored, smart technology initiatives can be developed, and focus can be applied to specific populations that have idiosyncratic needs. Connecting and collaborating with government departments has been cited as an enabling factor in bringing greater attention to mental health and mental health initiatives [22,23]. Lack of awareness has also been reported as a crucial barrier in advocating for public policy advocacy in mental health [24]. As such, studies into smart technology and eHealth should be regarded as transformative research in altering the landscape of how mental health care and support are provided and defined. Through the combination of policy and practice, smart technology innovations could find themselves as new methods of delivering cost-effective health care.

Crucial to the success of smart technology innovation is adherence to ethical guidelines. A literature review of eHealth revealed four key themes in regard to ethics; privacy, trust, beneficence and nonmaleficence, justice, and trust [25]. The intervention provided to participants was met with acceptability and positivity, with no issues concerning distrust or beliefs of unethical conduct. Access to such devices has previously been proposed as a key ethics issue in eHealth which also represents an economic problem [26]. This study’s use of commercially available devices, therefore, represents a cost-effective compromise that offers support and communication in the home. Healthcare providers also need to be trained and familiar with technological innovations in order to provide adequate eHealth care needed for their clients. Being unable to do so may result in further restrictions on access to care and/or isolation which would inhibit equity and justice for people with mental illness. Linked to the concept of privacy is the need for a stringent data security plan to develop trust with the technology and ensure that the rights of clients are protected. A key recommendation would be to engage stakeholders throughout the study process to examine and address risks that can assist in lessening unexpected ethical implications [27].

The COVID-19 pandemic meant lockdowns were enforced during the course of the study. Individuals with mental illness and physical conditions are especially vulnerable to the negative outcomes of isolation and distancing [28]. The COVID-19 pandemic meant that virtual and telephone interviews were conducted instead. Focus groups were largely conducted individually due to altered healthcare provider schedules and the inability of multiple people to meet in one place. Although participants were satisfied with these arrangements, some participants were difficult to contact and required multiple sessions to complete the interview. The technician for this study provided technical support where possible, but due to the COVID-19 pandemic and lockdown rules, opportunities to visit the participant and check the devices were limited.

Due to the small sample in this study, regression analyses were not conducted; thus, findings should be interpreted with consideration to this. However, the data collected from questionnaires were pertinent as it provided a better understanding of the characteristics of individuals with mental and physical health diagnoses who may benefit from smart technology use. Notably, it was observed through questionnaire scores that there was high heterogeneity in our sample and in participants’ responses (e.g., frequency of service utilization). Further research into the use of smart technology with individuals who have mental illness with a larger sample is warranted. All participants from this study were recruited from the same moderate-sized city. A larger study with a variety of locations, including more rural-based individuals, may reveal different experiences and learnings.

## 5. Conclusions

This study established a feasible and reliable way of informing future mental health care for people with comorbid physical conditions. As well this study provides a foundation for future research to expand upon and explore other smart home innovations using an Internet of Things approach. Commercially available devices were provided and implemented into the intervention, which offers an easily replicable approach compared to non-commercial medical devices. The intervention was also customizable, with devices selected or declined based on the needs of the participants, with input from their healthcare providers. As reported from the qualitative analyses, the technologies supported healthier lifestyles, both in terms of mental and physical health. Enhancements to communication were believed to have supported mental health and reduced the risks of isolation. The devices supplemented health benefits such as medication adherence and weight loss, as well as an increase in diet tracking and physical activity. In light of this, future researchers and funders should be enamored to pursue research with a high-risk and high-needs population. This study demonstrates worthwhile benefits and active engagement from vulnerable individuals as well as feasible intervention and methodology. Despite these findings, it is important to note that this study was conducted during the lockdowns of the COVID-19 pandemic, which may have impacted the findings. In particular, the health, social, and justice service utilization measures were likely affected and would need further study to investigate service usage more accurately. Addressing the gaps in the literature would provide greater evidence to assist changes to policy and the health care system as a whole. Future research could use larger samples to quantitatively examine conjectures generated from this study’s qualitative findings, such as the improvement of quality of life with the use of such technology by this population. It is recommended that policymakers and decision-makers explore the use of in-home technologies by providing devices to people with mental illness in the community. Doing so may allow for efficient communication and additional health benefits that will support people in their own homes and their health care providers.

## Figures and Tables

**Table 1:** Demographics (*N* = 13).

Age (mean (SD))	43 (15)
Sex	
Female	8
Male	5
Marital Status	
Single/Never Married	10
Separated/Divorced	2
Widowed	1
Housing Status	
Independent Apartment	9
Group Home	4
Psychiatric Diagnosis	
Anxiety Disorder	9
Mood Disorder	7
Psychotic Disorder	5
Disorder of Childhood/Disorder	3
Personality Disorder	3
Substance-related Disorder	1
Physical Diagnosis	
Fibromyalgia	3
Back Pain	2
Diabetes, Endometriosis, Hepatitis B, Hypertension, Irritable Bowel Syndrome, Peripheral Vasculitis, Polycystic Ovary Syndrome, Sleep Apnea, Ulcer (Foot)	1 for each

**Table 2:** Community Integration Mean Scores.

	Time 1 (*N* = 13) Mean (SD)	Time 2 (*N* = 11) Mean (SD)	Time 3 (*N* = 9) Mean (SD)
CIQ-R Total (/35) Missing (*n*)	20.7 (5.69)	21.7 (4.67)	21.6 (4.99)
	2	2	1
Home Integration (/12) Missing (*n*)	8.81 (2.25)	9.56 (1.61)	8.63 (2.25)
	1	2	1
Social Integration (/10) Missing (*n*)	5.92 (2.06)	6.00 (2.21)	5.78 (2.86)
	0	1	0
Productivity (/7) Missing (*n*)	2.33 (1.78)	2.27 (2.10)	2.22 (1.79)
	1	0	0
Electronic Social Networking (/6) Missing (*n*)	3.23 (1.48)	3.00 (1.95)	3.78 (1.79)
	0	0	0

**Table 3:** Short Form 36 Subscale Scores.

SF-35 Subscale	Time 1 (*N* = 13) Mean (SD)	Time 2 (*N* = 11) Mean (SD)	Time 3 (*N* = 9) Mean (SD)
Physical Functioning	53.1 (34.3)	51.7 (33.3)	53.9 (37.6)
Role Limitations due to Physical Health	51.9 (42.6)	47.5 (50.6)	36.1 (41.7)
Role Limitations due to Emotional Problems	46.2 (44.2)	23.3 (35.3)	55.6 (47.1)
Energy/Fatigue	43.5 (17.0)	34.3 (16.8)	49.4 (26.4)
Emotional Well-being	59.1 (19.2)	54.0 (21.9)	57.8 (21.6)
Social Functioning	52.9 (22.9)	46.3 (22.9)	59.7 (35.2)
General Health	41.9 (18.8)	38.5 (21.2)	44.4 (27.9)

**Table 4:** EQ-5D-3L Visual Analogue Scale Scores.

	Time 1 (*N* = 13) Mean (SD)	Time 2 (*N* = 11) Mean (SD)	Time 3 (*N* = 9) Mean (SD)
Overall Health	68.5 (12.7)	64.1 (15.8)	58.2 (26.6)
Mental Health	68.1 (15.2)	64.6 (22.0)	67.8 (17.2)
Physical Health	60.4 (16.6)	54.6 (18.5)	55.2 (29.3)

**Table 5:** Services Accessed at all Timepoints.

Services in Past Month	Response	Time 1 (*N* = 13)	Time 2 (*N* = 11)	Time 3 (*N* = 9)
Seen a healthcare or social service provider at their office	Yes	12	8	2
	No	1	3	7
	Sum	49	23	18
Talked on the phone with a healthcare or social service provider at their office	Yes	6	6	5
	No	7	5	4
	Sum	24	33	14
Visited by a healthcare or social service provider	Yes	6	3	2
	No	7	8	7
	Sum	69	19	41
Services in Past 6 Months				
Outpatient services at hospital	Yes	6	4	2
	No	7	6	7
	Declined	0	1	0
	Sum	18	28	10
Called Crisis Line	Yes	3	5	4
	No	10	5	5
	Declined	0	1	0
	Sum	2	192	22
	Yes	3	5	4
Visited by Crisis Team	No	10	5	5
	Declined	0	1	0
	Sum	-	180	10
Emergency Room Visits	Yes	7	8	4
	No	6	3	5
	Sum	22	40	12
Been in an Ambulance	Yes	6	7	3
	No	7	4	6
	Sum	10	15	6

**Table 6:** Devices used Most Frequently and Least Frequently.

KERRYPNX	Time 2 (*N* = 11)	Time 3 (*N* = 9)
Devices Used Most Frequently		
Fitbit	6	5
Smartphone	5	3
Medication Dispenser	2	1
Weight Scale	1	0
Devices Used Least Frequently		
Touch-Screen Monitor	8	6
Fitbit	2	1
Weight Scale	1	1
Smartphone	1	1

## Data Availability

We do not have permission from our Funder or Research Ethics Board to share the data freely. Data can only be shared with co-investigators and/or partners for analysis purposes. This was also stated in our Consent forms with participants. The authors would be open to future collaborations with other research teams where data could be shared as partners and added to agreements with the Funder and Research Ethics Board.
